# A Randomized Controlled Trial to Evaluate the Analgesic Effectiveness of Periarticular Injections and Pericapsular Nerve Group Block for Patients Undergoing Total Hip Arthroplasty

**DOI:** 10.3390/jpm14040377

**Published:** 2024-03-30

**Authors:** Bora Lee, Tae Sung Lee, Jaewon Jang, Hyun Eom Jung, Kwan Kyu Park, Yong Seon Choi

**Affiliations:** 1 Department of Anesthesiology and Pain Medicine, Severance Hospital, Anesthesia and Pain Research Institute, Yonsei University College of Medicine, 50-1 Yonsei-ro, Seodaemun-gu, Seoul 03722, Republic of Korea; 2 Department of Orthopedic Surgery, Severance Hospital, Yonsei University College of Medicine, 50-1 Yonsei-ro, Seodaemun-gu, Seoul 03722, Republic of Korea

**Keywords:** analgesia, arthroplasty, replacement, hip, nerve block

## Abstract

Pericapsular nerve group (PENG) block and periarticular injection (PAI) provide motor-sparing analgesia following hip surgery. We hypothesized that PAI offers non-inferior pain relief compared with PENG block in patients undergoing primary total hip arthroplasty (THA). In this randomized trial, 66 patients who underwent primary THA under spinal anesthesia were assigned to the PENG or PAI groups. The primary endpoint was the resting pain score 24 h postoperatively. The secondary endpoints included pain scores at rest and during movement at 6 and 48 h postoperatively, quadriceps strength at 24 h postoperatively, and opioid consumption at 24 and 48 h postoperatively. The mean difference in pain scores at rest between the two groups was 0.30 (95% confidence interval [CI], −0.78 to 1.39) at 24 h postoperatively. The upper 95% CI was lower than the non-inferiority margin, indicating non-inferior performance. No significant between-group differences were observed in the pain scores at 6 and 48 h postoperatively. Additionally, no significant differences in quadriceps strength and opioid consumption were observed between the two groups. The PAI and PENG blocks provided comparable postoperative analgesia during the first 48 h after primary THA. Further investigation is required to determine the optimal PAI technique and local anesthetic mixture.

## 1. Introduction

Effective pain management after total hip arthroplasty (THA) is essential for early rehabilitation, patient satisfaction, and functional recovery [1,2]. A combination of multimodal analgesia, periarticular injections (PAI), and peripheral nerve blocks is integral to perioperative pain management in total hip and knee arthroplasties [3,4]. Interventions such as PAI and single-shot nerve blocks effectively achieve early postoperative pain relief during the initial peak period of acute pain after THA [3,5]. Furthermore, using peripheral nerve blocks is linked to substantial enhancements in clinical outcomes, encompassing reductions in cardiac, pulmonary, and renal complications; thromboembolic events; and cognitive dysfunction [4].

Intraoperative PAI is an efficient and safe intervention for pain management after THA [6]. PAI reduces peripheral nociception by administering a local anesthetic into the tissues surrounding the surgical site. Its minimal effect on motor nerves renders it advantageous for promoting safe early ambulation [3,7]. PAI is quick and straightforward, requiring no special equipment, making it easy for orthopedic surgeons to administer directly at the surgery site. Unfortunately, a standardized administration method has yet to be developed.

Ultrasound-guided pericapsular nerve group (PENG) block is a new regional hip analgesia technique that targets the articular branches of the obturator, accessory obturator, and femoral nerves [8,9,10]. Previous studies reported that PENG blocks lower postoperative pain scores and opioid use as efficiently as fascia iliaca compartment block and preserves motor function better than fascia iliaca compartment block after THA [8,10]. The PENG block, aimed at deeper structures, is often performed using a curvilinear low-frequency ultrasound probe, unlike the linear probe for shallow structures, such as the femoral nerve, which requires more advanced skills [9]. While experienced anesthesiologists can perform a PENG block quickly, beginners might need more time. However, the PENG block’s standardized protocols ensures that its effectiveness remains largely consistent, regardless of the operator. 

To the best of our knowledge, a comparison of the analgesic effects of these two motor-sparing interventions on postoperative pain after THA is inadequate [11,12]. In this study, we hypothesized that PAI offers non-inferior analgesia compared to PENG block in patients undergoing primary THA.

## 2. Materials and Methods

This randomized controlled study was approved by the Severance Hospital Institutional Review Board (protocol number: 4-2021-0725; date of approval: 19 July 2021) and registered at ClinicalTrials.gov (NCT04981236; date of registration: 28 July 2021). This study followed the CONSORT guidelines for reporting clinical trials. A total of 74 adult patients scheduled to undergo elective primary THA were sequentially enrolled between August 2021 and May 2023. The exclusion criteria were allergy to local anesthetics, hepatic or renal insufficiency, coagulopathy, and major ipsilateral hip surgery history. Written informed consent was obtained from all the patients.

All patients underwent spinal anesthesia with a dose ranging from 10 to 12 mg of 0.5% hyperbaric bupivacaine. Throughout the surgical procedures, patients were positioned in the lateral decubitus posture, secured with a pelvic positioner, and operated on by a single surgeon employing the posterolateral hip approach. The short external rotators were then repaired. Cementless press-fit stems were used in all cases. No surgical drains were used postoperatively. All patients received preoperative oral celecoxib at a dose of 200 mg and intraoperative intravenous doses of acetaminophen (1 g), tranexamic acid (1 g), and dexamethasone (5 mg).

### 2.1. Administration of PENG Block and PAI

Patients were randomly allocated to receive the PENG block (PENG group) or PAI (PAI group) through a computerized random sequence generated by MedCalc Statistical Software (version 18.11.3; MedCalc Software Ltd., Ostend, Belgium). The assignment was overseen by a researcher who was not involved in assessing postoperative outcomes. 

A PENG block was performed with the patient in the supine position after closing the skin. The ultrasound transducer was positioned parallel to the inguinal ligament and rotated 45° to locate the iliopubic eminence, anteroinferior iliac spine, and the psoas tendon. Using the in-plane technique, a 22-gauge, 80 mm echogenic needle was inserted in a lateral-to-medial direction through the iliopsoas muscle until its tip reached the periosteum dorsal to the psoas tendon. After negative aspiration, a total volume of 20 mL of ropivacaine 0.3% with epinephrine 5 μg/mL was injected. 

PAI was performed using a total volume of 50 mL, comprising 50 mL of ropivacaine 0.3% mixed with 30 mg of ketorolac and epinephrine (6 μg/mL). The mixture was loaded into two 25 mL syringes at the beginning of surgery. After insertion of the acetabular component, the surgeon infiltrated the deep tissues (anterior and posterior capsules, gluteus minimus and medius muscles, and supraacetabular area) using the first 25 mL syringe. After insertion of the femoral stem, the gluteus maximus, abductors, tensor fascia lata, and subcutaneous tissues were infiltrated with a second 25 mL syringe. 

### 2.2. Postoperative Management

Each patient was administered oral celecoxib (200 mg) and intravenous acetaminophen (1 g) every 12 h as part of the postoperative pain management protocol. All the patients underwent postoperative exercise following the same rehabilitation protocol. After surgery, they performed bedside exercises, such as ankle pumps, quadriceps stretches, and leg raising exercises, within the first 6 h postoperatively. On postoperative day 1, standing and walking ambulation were allowed based on the same rehabilitation protocol. Rescue analgesia in the form of intravenous tramadol (50 mg) was administered to patients whose numeric rating scale (NRS) pain score exceeded 4. 

### 2.3. Outcome Assessments

The primary outcome measure was the resting pain score assessed 24 h after surgery. The secondary endpoints included pain scores at other times, opioid consumption, and muscle strength. Pain intensities at rest and during movement were assessed using an 11-point NRS (0 = no pain, 10 = worst imaginable pain) at four different time points: preoperative baseline and 6, 24, and 48 h after the surgery. Tramadol use was recalculated as an equivalent dose of oral morphine. [13]. Strength in the quadriceps of both legs was measured using a hand-held dynamometer (Lafayette Manual Muscle Test System, Lafayette Instrument Company, Lafayette, IN, USA) preoperatively and 24-h postoperatively [14]. Patients were instructed to perform knee extensions twice, with a 30-s interval between each attempt, and the analysis focused on the highest force achieved. In addition, perioperative data such as operation and anesthesia time, length of post-anesthesia care unit stay, Korean version of the Richards–Campbell Sleep Questionnaire (K-RCSQ), length of hospital stay, and preoperative and 3 and 6 months postoperative functional outcome scores (Western Ontario and McMaster Universities Osteoarthritis Index [WOMAC] and Harris Hip Score [HHS], respectively) were collected. The K-RCSQ was used to evaluate the sleep quality [15]. 

### 2.4. Statistical Analysis

Based on prior studies, the sample size was calculated assuming a 1.7 and 2.1 standard deviation of the pain score for PENG and PAI, respectively, at 24 h following primary THA [16,17]. The non-inferiority margin was 1.439, which was calculated using the fixed-margin method. To achieve a significance level of 2.5% and a power of 80%, 29 patients were needed for each arm of this study. Considering a dropout rate of 10%, 33 patients were enrolled in each group. The preoperative measurements and secondary outcomes were compared. Parametricity was determined using the Shapiro–Wilk and Kolmogorov–Smirnov tests. Parametric continuous variables were analyzed using the independent t-test, whereas non-parametric continuous variables were analyzed using the Mann–Whitney U test. Comparisons between groups for categorical data were performed using either Fisher’s exact test or the chi-square test, as appropriate. Continuous data are reported as the mean ± standard deviation for parametric measures and median (interquartile range) for non-parametric measures. Categorical data are presented as numbers (percentages). All statistical analyses were performed using R (version 3.5.1; R Foundation for Statistical Computing, Vienna, Austria), SPSS (version 23.0; IBM Corp., Armonk, NY, USA), and MedCalc Statistical Software (version 18.11.3; MedCalc Software Ltd., Ostend, Belgium). Statistical significance was set at *p* < 0.05.

## 3. Results

**Figure 1:**
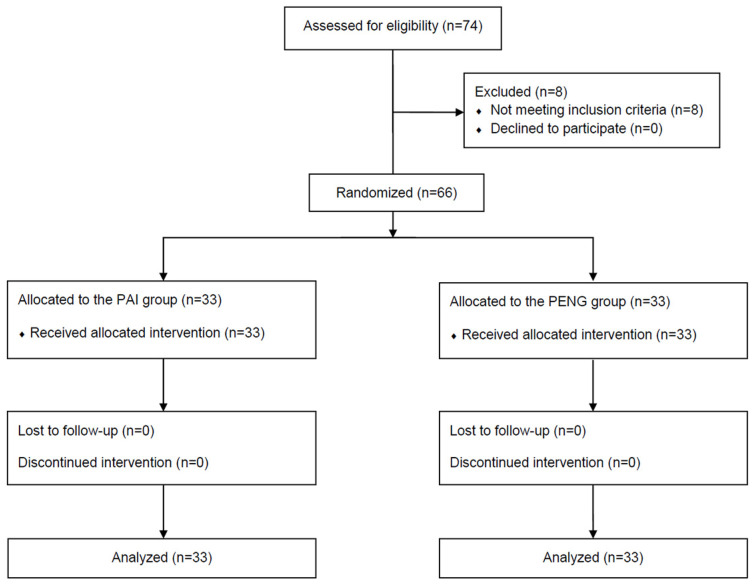
CONSORT study flow diagram. CONSORT, Consolidated Standards for Reporting Trials; PENG, pericapsular nerve group block; PAI, periarticular injection.

Among the 74 patients screened for eligibility, 66 were selected and assigned to the PAI or PENG groups. No dropouts were observed during the study period, and all the enrolled patients were included in the final analysis. Figure 1 presents the study flowchart.

No significant differences were identified between the two groups in terms of patient characteristics or operative data (Table 1).

**Figure 2:**
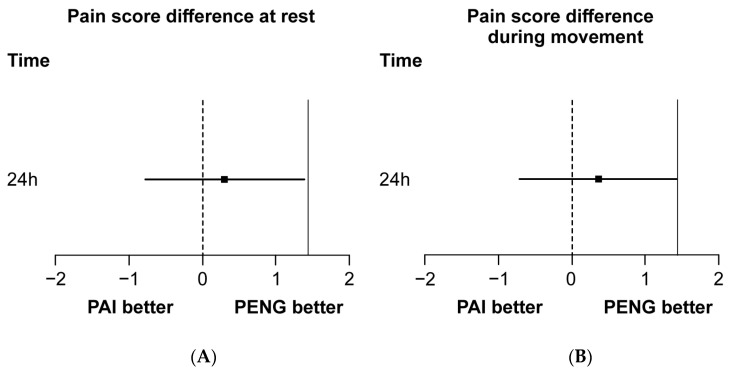
(**A**,**B**) Non-inferiority diagrams show the difference in the numerical rating scale pain score between the PENG and PAI groups 24 h postoperatively. The solid line indicates the non-inferiority margin (δ) of 1.439. Squares indicate mean pain score differences, and error bars indicate 95% confidence intervals of the difference between the groups. PENG, pericapsular nerve group block; PAI, periarticular injection.

The mean resting pain scores at 24 h postoperatively were 3.27 ± 2.39 and 2.97 ± 0.99 in the PAI and PENG groups, respectively, indicating a between-group difference of 0.30 (95% confidence interval [CI], −0.78 to 1.39). The upper limit of the 95% CI was below the non-inferiority margin (δ = 1.439), indicating a non-inferior performance (Figure 2A,B).

**Figure 3:**
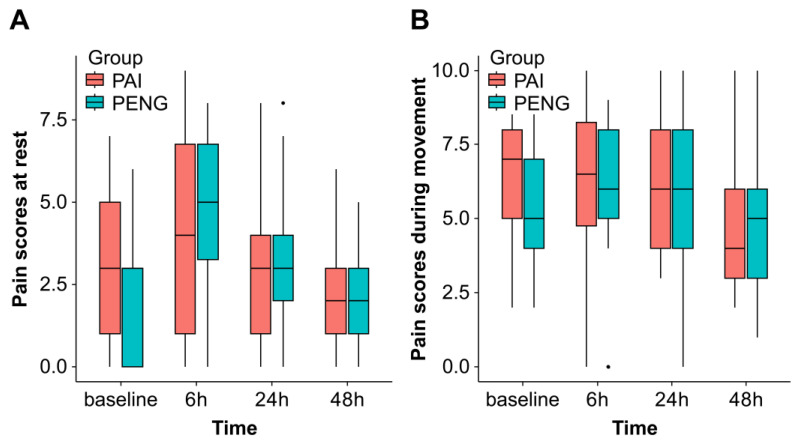
(**A**,**B**) Pain scores. Boxplots represent the median with 25th/75th percentiles. The whiskers show the minimum/maximum values, excluding outliers. Points represent outliers. PENG, pericapsular nerve group block; PAI, periarticular injection.

Similarly, the pain scores during movement at 24 h postoperatively did not show inferiority, with no statistically significant between-group differences. The mean resting pain scores at 6 and 48 h postoperatively were slightly lower in the PAI group compared with those in the PENG group (6 h: 3.9 vs. 4.6, mean difference, −0.69; 95% CI, −2.29 to 0.90; 48 h: 1.9 vs. 2.3, mean difference, −0.46; 95% CI, −1.25 to 0.33); however, there were no statistically significant differences. Additionally, no significant between-group differences were observed in pain scores during movement at 6 and 48 h postoperatively (Figure 3A,B).

No statistically significant difference was noted in quadriceps strength 24 h postoperatively (Table 2). The number of patients requiring rescue analgesics at 24 and 48 h postoperatively was comparable between the two groups. Similarly, no significant difference was observed in opioid consumption between the two groups at 24 and 48 h after surgery. The scores of the sleep quality profiles at 24 h after surgery were also comparable between the two groups. Moreover, the HHS and WOMAC scores were comparable between the two groups at 3 and 6 months postoperatively (Table 3). No postoperative falls were observed in any of the patients.

## 4. Discussion

In this randomized trial, the analgesic efficacy of PAI was compared with that of PENG block in patients undergoing primary THA. Our results showed that both strategies had similar analgesic effects on postoperative pain intensity during the first 48 h after THA using a posterolateral approach. Additionally, we observed that opioid consumption, quadriceps strength, and postoperative functional outcome scores did not differ between the two analgesic strategies.

PAI or peripheral nerve blocks are common analgesic strategies for pain management after THA; however, no intervention has yet been established as a standard analgesic strategy [3,6,7]. PAI, a procedure easily performed by surgeons, may offer advantages over peripheral nerve blocks for early ambulation [3,7]. This study compared the analgesic efficacy of PAI (50 mL 0.3% ropivacaine) with that of the PENG block (20 mL 0.3% ropivacaine), both of which have potential motor-sparing benefits and observed no differences in postoperative pain control and quadriceps strength. Recently, two studies compared the effectiveness of PAI and PENG blocks for THA using a posterolateral approach under spinal anesthesia [11,12]. Our findings are consistent with those reported by Zheng et al., who concluded that PAI (100 mL 0.15% ropivacaine) and PENG block (30 mL 0.5% ropivacaine) provided comparable analgesia [12]. Bravo et al.’s results differ from ours in that PAI was associated with lower static and dynamic pain scores in the first 24 h and 6 h after THA, respectively; however, their findings are consistent with ours in that the occurrence of quadriceps weakness did not differ between PAI (60 mL 0.25% bupivacaine) and PENG block (20 mL 0.5% bupivacaine) [11]. Two factors may explain the differences in the postoperative analgesic effects. First, compared with the standardized PENG block, the techniques for PAI vary according to the surgeon’s discretion, and the target tissues are not the same. Therefore, the analgesic effectiveness of PAI can differ depending on the technique used by individual operators. Second, PAI mixtures and perioperative multimodal analgesics varied between studies [11,12]. In addition, the PENG block requires an ultrasound device, a block needle, and additional sterile drapes, leading to higher costs than PAI. Despite its higher cost and longer preparation time, the PENG block offers similar or even inferior analgesic effects compared to PAI [11,12]. Consequently, in situations such as emergency surgeries or when rapid operating room turnover is needed and the PENG block is not feasible, PAI can provide effective analgesia for patients undergoing THA.

The innervation of the hip joint is relatively intricate [18,19,20]. Anatomical studies show that the anterior hip capsule and superior labrum have the greatest concentration of nociceptors and mechanoreceptors, primarily supplied by the femoral and obturator nerves [18,19,20]. Conversely, the posterior capsule has a relatively lower nociceptor density, which is innervated by the nerves to the quadratus femoris, articular branches of the sciatic nerve, and superior gluteal nerve [18,19]. In a recent anatomical study, the articular branches of the femoral nerve were effectively targeted by a PENG block using a single injection of 20–30 mL solution [21]. Interestingly, the spread of the dye to the femoral nerve was proportional to the volume of the injectate, consistent with the quadriceps weakness observed in clinical studies of PENG block [8,11,21]. However, the articular branches of the obturator nerve were seldom impacted, even with 30 mL of injectate [21]. Herein, we conducted a preliminary cadaveric trial to assess the pattern of dye spread following PAI. After capsulotomy and removal of the femoral head, PAI was performed by the surgeon on two fresh-frozen cadavers, excluding subcutaneous tissue infiltration, according to clinical study methods. Parts of the femoral nerve and nearly all articular branches of the femoral nerve were stained (Figure S1). However, the obturator nerve and its articular branches were rarely stained (Figure S2), and the superior gluteal nerve was stained (Figure S3). According to anatomical studies, the superior gluteal nerve possesses a small articular branch associated with posterior hip innervation; however, this finding is not consistently observed [19,22]. Taking into account these anatomical findings, PAI may offer advantages over the PENG block in postoperative pain management after THA as it potentially addresses pain in both the anterior and posterior capsules. However, further anatomical and radiological studies analyzing dye spread patterns in cadavers are necessary to establish a standardized PAI technique for THA.

While this study uncovered significant insights regarding the efficacy of PAI, it also had several limitations. First, blinding was not optimal because the patients did not receive a sham PENG block or PAI. Second, our results are specific to the adjuvants, concentration, and total volume of the local anesthetic used for the PAI and PENG blocks. Finally, in a preliminary cadaveric trial, we observed that almost all articular branches of the femoral nerve were affected by PAI; however, the small number of cadavers made it difficult to draw generalized conclusions. Additionally, because PAI was performed in the lateral position and dissections were conducted in the supine and prone positions, dye dispersion and postmortem changes in tissue integrity and permeability may have been affected.

In conclusion, the PAI and PENG blocks provided comparable postoperative analgesia during the first 48 h after primary THA. Additional clinical and anatomical studies are necessary to identify the optimal technique and local anesthetic regimen for PAI in THA.

## Figures and Tables

**Table 1:** Patient characteristics and operative data.

	PAI Group (N = 33)	PENG Group (N = 33)	*p*-Value
Demographic data			
Age (years)	60.0 (50.0–70.0)	62.0 (56.0–67.0)	0.792
Sex (Female/Male)	17/16	17/16	>0.999
Height (cm)	163.8 ± 10.2	162.6 ± 8.0	0.620
Weight (kg)	65.5 ± 9.3	65.2 ± 9.7	0.909
Body mass index (kg/m^2^)	24.4 ± 2.9	24.6 ± 2.7	0.830
ASA class (I/II/III)	9/16/8	7/20/6	0.613
Length of surgery (min)	67.1 ± 12.3	66.9 ± 16.2	0.952
Anesthesia time (min)	107.3 ± 20.5	106.5 ± 19.9	0.879
Blood loss (mL)	100.0 (50.0–100.0)	100.0 (50.0–135.0)	0.581

Values are presented as the median (interquartile range), mean ± standard deviation, or number of patients. ASA, American Society of Anesthesiologists; PENG, pericapsular nerve group block; PAI, periarticular injection.

**Table 2:** Quadriceps strength, opioid consumption, and sleep profiles.

	PAI Group (N = 33)	PENG Group (N = 33)	*p*-Value
Quadriceps strength of the operative leg (kgf)			
Preoperative	9.8 (7.3–12.2)	10.5 (8.2–13.8)	0.190
Postoperative 24 h	6.8 (5.5–8.9)	6.2 (5.2–9.2)	0.740
Quadriceps strength of the non-operative leg (kgf)			
Preoperative	12.2 ± 3.3	12.2 ± 3.2	0.928
Postoperative 24 h	11.4 ± 3.1	11.5 ± 3.7	0.879
Patients requiring rescue analgesics (n)			
0–24 h	15 (46%)	19 (58%)	0.460
24–48 h	13 (39%)	10 (30%)	0.605
0–48 h	17 (52%)	19 (58%)	0.805
Cumulative opioid consumption (morphine equivalents)			
0–24 h	0 (0–0.3)	0.2 (0–0.3)	0.525
24–48 h	0 (0–0.2)	0 (0–0.2)	0.547
0–48 h	0.2 (0–0.6)	0.2 (0–0.4)	0.844
Sleep quality profiles			
Sleep depth	30.0 (20.0–65.0)	40.0 (20.0–70.0)	0.556
Sleep latency	60.0 (30.0–60.0)	40.0 (20.0–80.0)	0.629
Awakening	40.0 (20.0–55.0)	40.0 (20.0–70.0)	0.601
Returning sleep	50.0 (25.0–70.0)	55.0 (20.0–80.0)	0.676
Sleep quality	40.0 (10.0–60.0)	50.0 (20.0–70.0)	0.376

Values are presented as median (interquartile range), mean ± standard deviation, or number of patients (%). PENG, pericapsular nerve group block; PAI, periarticular injection.

**Table 3:** Postoperative outcomes.

	PAI Group (N = 33)	PENG Group (N = 33)	*p*-Value
Harris Hip score			
Preoperative	49.0 ± 19.7	51.1 ± 21.0	0.685
Postoperative 3 months	88.0 (81.9–96.0)	92.0 (84.0–95.0)	0.517
Postoperative 6 months	91.7 (88.0–96.0)	89.5 (84.3–95.0)	0.545
WOMAC score			
Preoperative	53.3 ± 16.1	48.2 ± 17.6	0.227
Postoperative 3 months	20.5 ± 9.7	21.5 ± 13.6	0.779
Postoperative 6 months	18.9 ± 12.7	21.8 ± 11.8	0.479

Values are presented as the median (interquartile range) or mean ± standard deviation. PENG, pericapsular nerve group block; PAI, periarticular injection; WOMAC, Western Ontario and McMaster Universities Arthritis Index.

## Data Availability

The data supporting the findings of this study are available upon request from the corresponding author.

## Supplementary Materials

The following supporting information can be downloaded at: https://www.mdpi.com/article/10.3390/jpm14040377/s1, Figure S1: Articular branches of the femoral nerve (red arrow) are colored with the dye after periarticular injection. Figure S2: Articular branches of the obturator nerve (blue arrow) are rarely stained with the dye after periarticular injection. Figure S3: Superior gluteal nerve (red arrow) is colored with the dye after periarticular injection.

## References

[1] Bober K., Kadado A., Charters M., Ayoola A., North T. (2020). Pain control after total hip arthroplasty: A randomized controlled trial determining efficacy of fascia iliaca compartment blocks in the immediate postoperative period. J. Arthroplast..

[2] Højer Karlsen A.P., Geisler A., Petersen P.L., Mathiesen O., Dahl J.B. (2015). Postoperative pain treatment after total hip arthroplasty: A systematic review. Pain.

[3] Wainwright T.W., Gill M., McDonald D.A., Middleton R.G., Reed M., Sahota O., Yates P., Ljungqvist O. (2020). Consensus statement for perioperative care in total hip replacement and total knee replacement surgery: Enhanced Recovery after Surgery (ERAS(^®^)) Society recommendations. Acta Orthop..

[4] Memtsoudis S.G., Cozowicz C., Bekeris J., Bekere D., Liu J., Soffin E.M., Mariano E.R., Johnson R.L., Go G., Hargett M.J. (2021). Peripheral nerve block anesthesia/analgesia for patients undergoing primary hip and knee arthroplasty: Recommendations from the International Consensus on Anesthesia-Related Outcomes after Surgery (ICAROS) group based on a systematic review and meta-analysis of current literature. Reg. Anesth. Pain Med..

[5] Panzenbeck P., von Keudell A., Joshi G.P., Xu C.X., Vlassakov K., Schreiber K.L., Rathmell J.P., Lirk P. (2021). Procedure-specific acute pain trajectory after elective total hip arthroplasty: Systematic review and data synthesis. Br. J. Anaesth..

[6] Ma H.H., Chou T.A., Tsai S.W., Chen C.F., Wu P.K., Chen W.M. (2019). The efficacy of intraoperative periarticular injection in total hip arthroplasty: A systematic review and meta-analysis. BMC Musculoskelet. Disord..

[7] Jiménez-Almonte J.H., Wyles C.C., Wyles S.P., Norambuena-Morales G.A., Báez P.J., Murad M.H., Sierra R.J. (2016). Is local infiltration analgesia superior to peripheral nerve blockade for pain management after THA: A network meta-analysis. Clin. Orthop. Relat. Res..

[8] Aliste J., Layera S., Bravo D., Jara Á., Muñoz G., Barrientos C., Wulf R., Brañez J., Finlayson R.J., Tran Q. (2021). Randomized comparison between pericapsular nerve group (PENG) block and suprainguinal fascia iliaca block for total hip arthroplasty. Reg. Anesth. Pain Med..

[9] Girón-Arango L., Peng P.W.H., Chin K.J., Brull R., Perlas A. (2018). Pericapsular nerve group (PENG) block for hip fracture. Reg. Anesth. Pain Med..

[10] Choi Y.S., Park K.K., Lee B., Nam W.S., Kim D.H. (2022). Pericapsular nerve group (PENG) block versus supra-inguinal fascia iliaca compartment block for total hip arthroplasty: A randomized clinical trial. J. Pers. Med..

[11] Bravo D., Aliste J., Layera S., Fernández D., Erpel H., Aguilera G., Arancibia H., Barrientos C., Wulf R., León S. (2023). Randomized clinical trial comparing pericapsular nerve group (PENG) block and periarticular local anesthetic infiltration for total hip arthroplasty. Reg. Anesth. Pain Med..

[12] Zheng L., Jo Y., Hwang J., Rhim H., Park E., Oh C., Lee J., Noh C., Hong B., Lee J. (2022). Comparison of the analgesic efficacy of periarticular infiltration and pericapsular nerve group block for total hip arthroplasty: A randomized, non-inferiority study. Ann. Palliat. Med..

[13] Nielsen S., Degenhardt L., Hoban B., Gisev N. (2016). A synthesis of oral morphine equivalents (OME) for opioid utilisation studies. Pharmacoepidemiol. Drug Saf..

[14] Maffiuletti N.A. (2010). Assessment of hip and knee muscle function in orthopaedic practice and research. J. Bone Jt. Surg. Am..

[15] Kim J.K., Park J.H., Cho J., Lee S.M., Lee J. (2020). Reliability of the Korean version of the Richards-Campbell Sleep Questionnaire. Acute Crit. Care.

[16] Kurosaka K., Tsukada S., Ogawa H., Nishino M., Yoshiya S., Hirasawa N. (2020). Comparison of early-stage and late-stage periarticular injection for pain relief after total hip arthroplasty: A double-blind randomized controlled trial. J. Arthroplast..

[17] Pascarella G., Costa F., Del Buono R., Pulitanò R., Strumia A., Piliego C., De Quattro E., Cataldo R., Agrò F.E., Carassiti M. (2021). Impact of the pericapsular nerve group (PENG) block on postoperative analgesia and functional recovery following total hip arthroplasty: A randomised, observer-masked, controlled trial. Anaesthesia.

[18] Simons M.J., Amin N.H., Cushner F.D., Scuderi G.R. (2015). Characterization of the neural anatomy in the hip joint to optimize periarticular regional anesthesia in total hip arthroplasty. J. Surg. Orthop. Adv..

[19] Laumonerie P., Dalmas Y., Tibbo M.E., Robert S., Durant T., Caste T., Vialla T., Tiercelin J., Gracia G., Chaynes P. (2021). Sensory innervation of the hip joint and referred pain: A systematic review of the literature. Pain Med..

[20] Ross J.A., Greenwood A.C., Sasser P., Jiranek W.A. (2017). Periarticular injections in knee and hip arthroplasty: Where and what to inject. J. Arthroplast..

[21] Kim J.Y., Kim J., Kim D.H., Han D.W., Kim S.H., Kim D., Chung S., Yu S., Lee U.Y., Park H.J. (2023). Anatomical and radiological assessments of injectate spread stratified by the volume of the pericapsular nerve group block. Anesth. Analg..

[22] Nagpal A.S., Brennick C., Occhialini A.P., Leet J.G., Clark T.S., Rahimi O.B., Hulk K., Bickelhaupt B., Eckmann M.S. (2021). Innervation of the posterior hip capsule: A cadaveric study. Pain Med..

