# Supplementary figures and images for: A Randomized Controlled Trial to Evaluate the Analgesic Effectiveness of Periarticular Injections and Pericapsular Nerve Group Block for Patients Undergoing Total Hip Arthroplasty
Source: J Pers Med. 2024 Mar 30;14(4):377. doi: 10.3390/jpm14040377 (PMC11051520; doi:10.3390/jpm14040377)

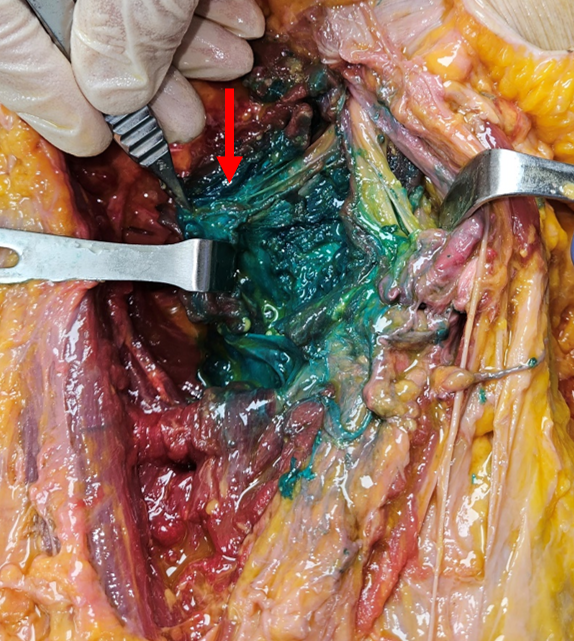

Supplement: Supplementary file 1 [file jpm-14-00377-s001.zip › supplemental figure S1.tif]

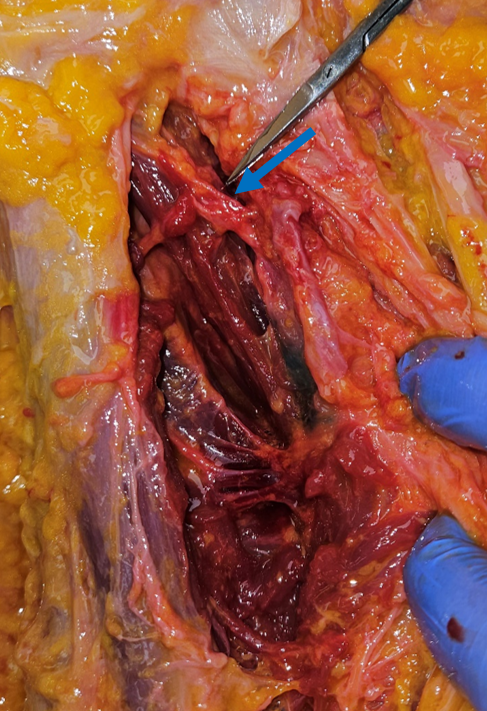

Supplement: Supplementary file 1 [file jpm-14-00377-s001.zip › supplemental figure S2.tif]

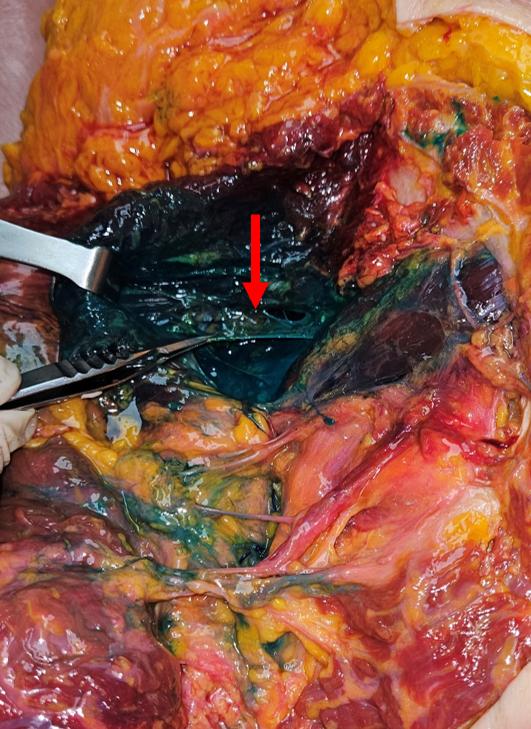

Supplement: Supplementary file 1 [file jpm-14-00377-s001.zip › supplemental figure S3.tif]
